# Algorithms for matching partially labelled sequence graphs

**DOI:** 10.1186/s13015-017-0115-y

**Published:** 2017-09-25

**Authors:** William R. Taylor

**Affiliations:** Francis Crick Institute, 1 Midland Road, London, NW1 1AT UK

**Keywords:** Phylogenetic tree matching, Correlated substitution analysis, Bipartite graph matching

## Abstract

**Background:**

In order to find correlated pairs of positions between proteins, which are useful in predicting interactions, it is necessary to concatenate two large multiple sequence alignments such that the sequences that are joined together belong to those that interact in their species of origin. When each protein is unique then the species name is sufficient to guide this match, however, when there are multiple related sequences (paralogs) in each species then the pairing is more difficult. In bacteria a good guide can be gained from genome co-location as interacting proteins tend to be in a common operon but in eukaryotes this simple principle is not sufficient.

**Results:**

The methods developed in this paper take sets of paralogs for different proteins found in the same species and make a pairing based on their evolutionary distance relative to a set of other proteins that are unique and so have a known relationship (singletons). The former constitute a set of unlabelled nodes in a graph while the latter are labelled. Two variants were tested, one based on a phylogenetic tree of the sequences (the topology-based method) and a simpler, faster variant based only on the inter-sequence distances (the distance-based method). Over a set of test proteins, both gave good results, with the topology method performing slightly better.

**Conclusions:**

The methods develop here still need refinement and augmentation from constraints other than the sequence data alone, such as known interactions from annotation and databases, or non-trivial relationships in genome location. With the ever growing numbers of eukaryotic genomes, it is hoped that the methods described here will open a route to the use of these data equal to the current success attained with bacterial sequences.

**Electronic supplementary material:**

The online version of this article (doi:10.1186/s13015-017-0115-y) contains supplementary material, which is available to authorized users.

## Background

### Introduction

The analysis of large multiple sequence alignments to reveal positions that co-vary (correlated mutations or substitutions) has recently become a powerful method to identify pairs of interacting positions that can be used as constraints in the construction of molecular models (see [1] for a review).

Correlated substitution analysis can also be used to find pairs of interacting positions between proteins if the multiple sequence alignments for two or more proteins are concatenated and processed as a single joint alignment. For this to work, however, requires that each pair of concatenated sequences coexist in the same organism (or a close relative) and have been subject to mutual evolutionary selection pressures.

If the pair of proteins is unique to that organism, then pairing-up the proteins on the basis of species name will produce the correct assignment. However, given the dominant mechanism of gene duplication and diversification in protein evolution, the more typical situation is that there will be a number of choices (paralogs) for each protein with no guidance from the species name to which pairs of proteins interact.

In bacteria, the genes of proteins that interact are often co-expressed and found close in the genome sequence on an operon in which all the genes are under common expression control. Therefore co-location on the genome can provide a good guide to help match pairs of proteins. A way to do this is simply to note the difference in the gene identifier that are assigned sequentially along the genome.

However in eukaryotes, gene expression control is complex and co-location does not imply co-expression or interaction of the resulting proteins. Indeed, interacting proteins can easily be located on different chromosomes.

### Outline of the approach

Without any simple aid to pairing proteins, the relative sequence similarity between the paralogous proteins can be used as a rough guide. For example, if protein A and a interact and both duplicate to produce B and b which at a later time duplicate again into C and c  then their phylogenetic tree will be:

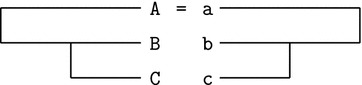



This structure would indicate that A/a is ancestral but remains agnostic on the pairing of the B/b and C/c proteins. (Note that no information can be obtained from comparing the sequences between the different families as these will generally be completely unrelated proteins).

The way in which the inclusion of the B/b, C/c proteins identified A/a as ancestral can be extended by the inclusion of more family members and a recent duplication of C/c to D/d might allow B/b to be identified as a matching pair. However, as the number of paralogs cannot generally be guaranteed to be large, a more general approach is needed. Indeed, as a result of an ancient double genome duplication in the common ancestor of the metazoa (*circa.* 500M years ago), the number of paralogs for any gene is typically four.

The sketch trees drawn above are unrooted and to provide a root, it is common in phylogenetic analysis to include a distant family member to establish a root to the tree (called the outgroup). A similar approach can be used to help in the current situation but with the condition that the ’outgroups’ should be drawn from a pool of related sequences that are unique to each species (and need not necessarily be distant relatives). For example, given three species: bat, cat and rat, the cat has four paralogs in each family (C1…4 and CA…CD) but the rat and bat have only one (B0, R0 and BZ, RZ). Including these extra sequences can resolve the ambiguous assignment of the cat sequences:

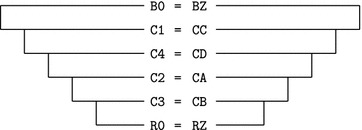



The known equivalence of the bat and rat sequences thus provides a reference frame allowing the relationships of all the cat proteins to be established.

Such a scheme would be sufficient to solve the current problem, even with just one ‘outgroup’ species were it not for the introduction of evolutionary ‘noise’. Substantial variation in the relationships between the sequences in each family are to be expected as each family (C1..4 and CA..D) are completely different proteins with different pressures on their selection and possibly even different duplication times if one interaction partner begins by ‘moonlighting’ with two others. In addition, the use of the outgroup species assumes that they are unique, whereas it may simply be that they have other, unsequenced or unidentified, partners.

To circumvent the limitation of evolutionary ‘noise’ and the uncertainty in outgroup uniqueness, the procedure can be repeated using different outgroups and different numbers of outgroups, building-up a consensus relationship among the paralogs. Ultimately, it would be best to use all the singleton outgroups to establish the relationships among the unlabeled sequences but the comparison of large trees is a computationally complex and demanding calculation. Instead, a method will be developed below as a pre-filter that uses just the distances between sequences, thus avoiding the complexity of matching tree topologies.

In addition, the procedure is not limited to a single pair of families and other families can be included to establish consistent (transitive) relationships among them. In general, given a set of proteins and species: 
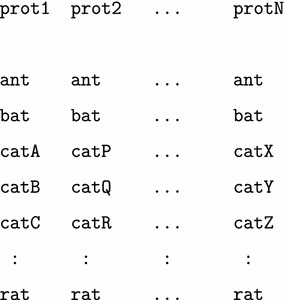
 sets of overlapping triples could be extracted and analysed for transitivity in their predicted matchings.

### Relationship to other methods

The approach outlined above is not unlike the mirror-tree method [2] that attempts to find similarity between two, often quite large, trees of (labelled) orthologues from two species with the aim to identify interacting proteins. While this method was considered for use in solving the current problem, it was avoided in favour of the simpler tree-matching methods in the PHYLIP package [3] which are sufficient for the smaller trees of paralogs.

The problem of paralog matching has recently been approached more directly by two similar methods that maximise the strength of the predicted contacts while iteratively introducing paralog pairings [4, 5]. Although only tested on a few examples, these methods give encouraging results but are limited by the heavy cost of continually recomputing the contacts. By contrast, the method developed below considers only direct sequence similarity which is a relatively fast calculation.

## Methods

### Topology-based algorithm

The comparison of phylogenetic trees outlined in the Introduction to discriminate unlabelled sequences in a partially labelled tree, relies on the relative distance of the unlabelled to the labelled sequences. Such a distance can only be computed over branch-lengths when the two trees are topologically identical and given the degree of divergence expected between trees based on completely different proteins, such isomorphism cannot be guaranteed or even expected. To circumvent this problem, a degree of ‘noise’ was added to the sequence distances and matching trees extracted from the variations thus generated. For these calculations, the PHYLIP package [3] was used: firstly the program protdist calculated the inter-sequence distances that were passed to the treedist program to find identical tree topologies. These were then re-processed by treedist using the branch lengths to return the distance between the trees (see “Implementation details” section, “PHYLIP package” for parameter settings).

This process was repeated a number of times and for different outgroup selections (see “Implementation details” section, “Outgroup selection and sequence distance scaling”). The pairings generated for the unlabelled nodes for each run were pooled and a final consensus generated using bipartite graph matching—as previously employed in the matching of unlabelled secondary structures in protein structure comparison [6]. (See “Implementation details” section, “Bipartite graph matching” for a description of the algorithm.) The resulting pairings were then used to produce the concatenated sequence alignment required for the analysis of inter-protein residue correlation (“Residue covariation analysis” section). The method was evaluated using different numbers of random trials, different numbers of outgroups and varying levels of random noise added to the distances. (Defined in more detail below).

The method is based on a pair of protein families, however, if three families are simultaneously considered then pairings that give consistent (transitive) solutions can be given precedence. As the method is stochastic, there may be solutions that fall short of transitivity within the ’noise’ level. So that these were not missed, a number of pairings were generated by adding a small amount of noise to the similarity matrix before it was parsed by the bipartite matching algorithm. The pairings from these randomised comparisons were re-summed into a score matrix and those that generated a consistent relationship across the pairs of families were up-weighted by a factor of ten. The final consensus matrix was then re-processed by the bipartite matching algorithm with the resulting final solution thus including a bias towards transitivity.

**Fig. 1:**
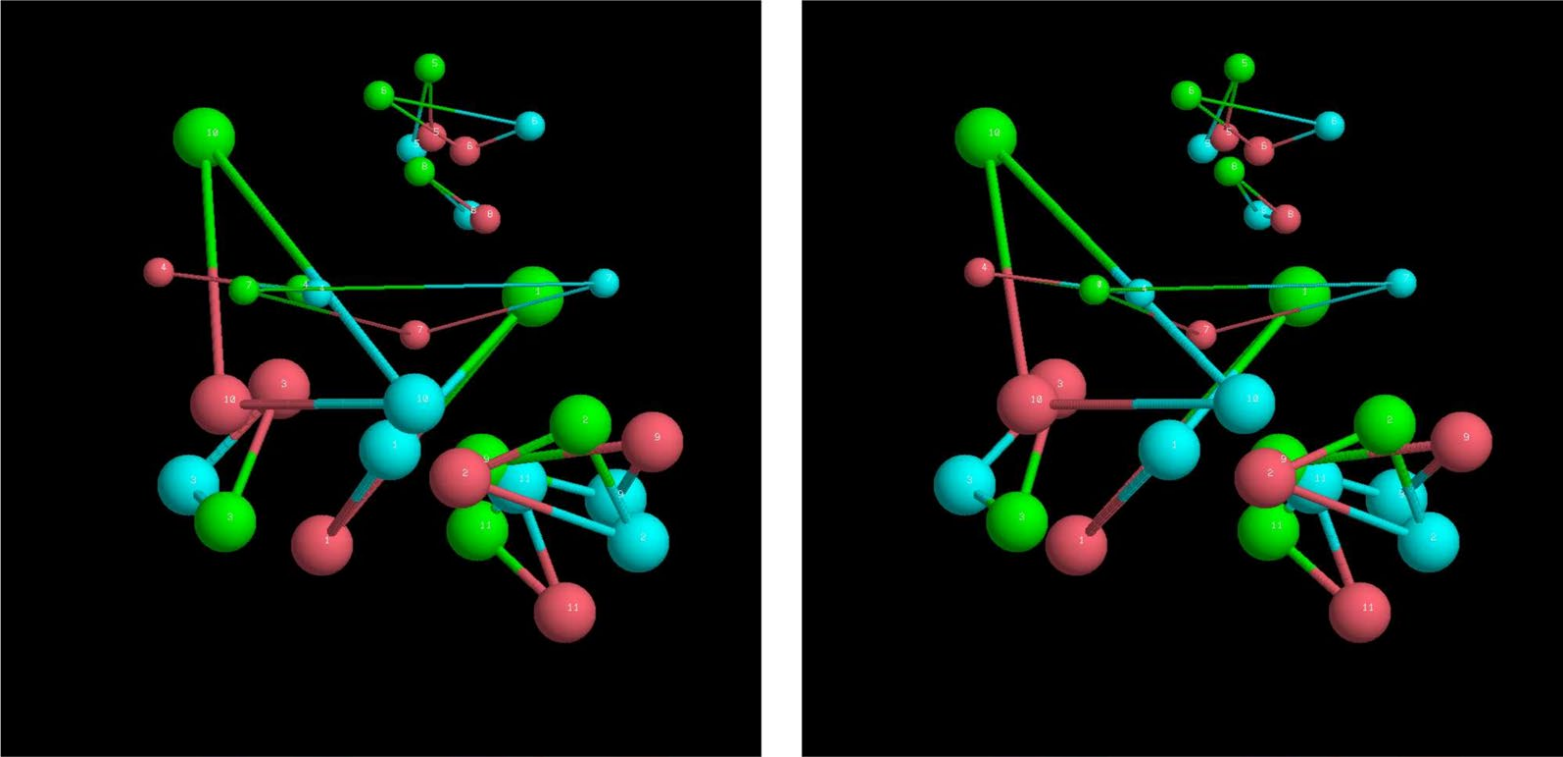
Sequence space visualisation. The distances between five unlabelled sequences (small balls) from three proteins (red, green, blue) are embedded in a sequence-space along with six ‘outgroups’ (larger balls). The known matching between the singleton outgroups is indicated by thick lines. The unknown matching to be predicted for the unlabelled sequences (parlogues) is indicated by fine lines. The two parts are a stereo-pair (to be viewed cross-eyed)

The quality of data that is processed is shown in Fig. 1 for five sequences from three proteins with six outgroups embedded in a sequence-space using a dimensional reduction method [7]. As no distances are known between the proteins, the three separate protein point-sets (coloured red, green, blue) were combined to have a minimum RMSD superposition of their outgroups (larger balls).

### Distance-based algorithm

As the number of sequences increases, the chance of finding matching topologies using the method described above becomes smaller, requiring a corresponding increase in computation time to gain success. In addition, each protein may have different numbers of sequences which adds to the complexity. To alleviate these difficulties, a simpler method was developed based only on the inter-sequence distances, without the constraint of representing these in a tree structure. This approach also allows the distances of the unlabeled sequences to all the singleton sequences to be used. In practice, however it was found that a few hundred is more than ample and 50 (default) is sufficient. The code will function with as few as two singletons but this limit was not tested.

The same method was followed as above, with the distances of each sequence to its intra-family singletons being compared between families to generate a matrix of similarities that can be processed by a bipartite graph-matching algorithm. To retain the capacity to apply the transitivity bias described above, the protein families were processed as sets of triplets with the bipartite algorithm operating on the three-dimensional matrix of similarities (strictly, now a tripartite matching algorithm). The resulting matches are thus reduced to a set of sequences equal to the size of the smallest family—as required for the tree-based algorithm described above.

As much of the same code and data were used for both methods, the inclusion of a few outgroups was also retained in the sequence set for the distance-based algorithm. Although, now redundant (as all singleton outgroups are compared), these additional sequences provided an internal control and only solutions, over a number of trials, that preserved the known identity of the outgroups were kept. As above, these were accumulated and re-processed by a final pass of the tripartite algorithm to produce a consensus matching.

### Solvent exposure filter

Structural information is assumed to be available for each protein, either in the form of a solved structure or as a predicted model based on calculated intra-sequence correlated mutation analysis, or any combination for a given set of proteins.

The known or model protein structures can add information on the likely veracity of any given predicted inter-molecular constraint. Most obviously, if an inter-protein contact is predicted between two buried residues, then it is less likely that the predicted contact is correct. (Although, the predicted pair may have co-evolved for reasons other than direct interaction.)

As any available structure must represent every sequence in a large alignment, the full-atom coordinates were not used to calculate the degree of burial for each position and as the structure may also be a relatively ’rough’ model, consisting only of $\alpha$-carbon coordinates, a pseudo-centroid was calculated for each side-chain [8] and the solvent accessible surface area (SASA) [9] calculated over this model using a probe radius of 5 Å, which is large enough to prevent cavities in the model appearing to be exposed.

The score of each pairwise contact was down-weighted in proportion to the product of the logs of their SASA exposure, as: $w = 1-\exp (-A_i \cdot A_j/100)$, where *A* is the SASA of each residue in the pair *i*, *j*. The spread factor of 100 (*c.f.* variance in the Normal distribution) was chosen empirically to exclude only pairs of residues where one or both positions was substantially buried. The weight has a range 0..1 which allows the SASA contribution to be factored in as a linear contribution by a factor *f* as: $S = f \cdot s + (1-f) \cdot w \cdot s$, where *s* is the covariance score for the pair. When $f=0$ there is no SASA contribution and when $f=1$ the full SASA weight is applied.

### Implementation details

#### PHYLIP package

Version 3.696 of the PHYLIP package was used [3]. (To download the package go to: http://evolution.genetics.washington.edu/phylip.htm).

The program protdist, which calculates a distance between two sequences, was used with the JTT matrix [10] and otherwise default parameters.

The program neighbor was used with the Neighbor-Joining method [11] to generate unrooted trees (taking the species in randomised order).

The program treedist was run in two modes: firstly with the “Symmetric Difference” option which uses the algorithm of Robinson and Flouds [12] to compare tree topologies, allowing those with an identical topology to be selected. However, as the PHYLIP programs only operate with labelled sequences, a ‘dummy’ working label was assigned to each sequence and all permutations of the working labels were tested allowing pairs of trees to be selected that had both identical topology and matching working labels. The difference between topologically identical trees was then calculated using the “Branch Score Distance” option which compares the lengths of the branches using the algorithm of Kuhner and Felsenstein [13].

#### Outgroup selection and sequence distance scaling

The selection of outgroups for the topology-based algorithm was made by random selection of a pair of sequences/species from the list of singletons. As these have a known correspondence across all protein sets, it is possible to check that they have a consistent relationship. The visual equivalent in Fig. 1 is to measure how small the triangles are that connect the large balls (that represent in this example, six outgroups from three proteins, coloured red, green, and blue). However, as the outgroup sets are not superposed as in Fig. 1, a proxy for this relationship is whether the intra-protein outgroup distances are similar between proteins.

As more than one outgroup is always chosen, the consistency of their inter-relationship can be tested by comparing the RMS deviation between equivalent pairs of outgroups across different proteins. Specifically, if $d_{ij}$ is the inter-sequence distance (calculated by the PHYLIP program protdist) between two outgroups, then over each set of outgroup sequences, an average distance was calculated as: $\bar{d} = \frac{1}{N}\sum d_{ij}$, over *N* pairs of sequences, and from this the RMSD ($\sigma$) was calculated as: $\sigma = \sqrt{(}\frac{1}{N}\sum (d_{ij} - \bar{d})^2)$. If $100\cdot \sigma$ fell above a given cutoff (initial value of $50n+100$, where *n* is the number of outgroups), then the selection was discarded and new outgroups were picked. However, to ensure some success, the cutoff was gradually increased by 10 with every selection.

As each set of sequences can come from completely unrelated proteins, these may have different rates of evolution making the comparison of internal distances (or branch-lengths) between protein sets less reliable. To reduce this effect, sequence distances were normalised using the average outgroup distance, $\bar{d}$, as calculated above, with the inter-sequence distance, $d_{ij}$ (for sequence pair *i*, *j*) replaced by: $d_{ij} \cdot 10/\bar{d}$.

#### Bipartite graph matching

Although the “Stable marriage” algorithm was used previously [6] it does not guarantee the highest score on a weighted graph and a simpler semi-greedy algorithm was implemented instead. This took the ranked list of edges and starting with the highest, generated a matching by working down the list of edges. To prevent the strongest edge dominating the solution, this was repeated starting with the 2nd, 3rd,… Nth edge in the rank, with N being the number of nodes in each set. All the edges above and below the starting edge were still allowed to be part of the solution and the solution with the highest sum of weighted edges was selected. These algorithms can be used with unequal number of nodes in the sets.

The same algorithm was used for tripartite matching but with a list of edges taken from all combinations of triple matches. Within a species, the inter-protein distances (*d*) were scaled and stored in the range 0...255 and between species *i* and *j*, a similarity measure (*s*) was calculated as: $s_{ij} = \exp ((d_i-d_j)^2/100)$ The entry in the matrix of matches for each triple of sequences (*i*, *j*, *k*) was then: $D_{ijk} = s_{ij}+s_{jk}+s_{ik} + r/10$, where *r* is a random number between 0 and 1.

The process was repeated and for each solution, the pair of labelled ‘outgroups’ was used as an internal quality-control check and only solutions that correctly matched these was accepted.

#### Sequence data

Sequence searches were made using the Hmmer method [14] using the server at the EBI (https://www.ebi.ac.uk/Tools/hmmer/search/phmmer) with the default search parameters. As some of the proteins have very large numbers of sequences, their number was limited to around 10,000 by using a variation of the representative proteomics database (pr15...pr75).

Some of the proteins have individual Pfam entries for each of their domains and they were used where available [15].

Species identity was established by the coded-identifier included in the SwissProt code (the part following the underscore). If only the full species name is available, this can also be used after the removal/replacement of spaces and concatenation to the sequence identifier using the underscore character.

A list of species codes can be found at: http://www.uniprot.org/docs/speclist. Only real species were used and the higher taxonomic groupings or “virtual species” (which have a code starting with ’9’) were excluded.

#### Residue covariation analysis

In previous studies, the GREMLIN method [16] had been found to give good quality contact predictions [17, 18] and also has the benefit of generating a best structure match to the predicted contacts (based on an iterated double-dynamic programming algorithm [19]). (http://gremlin.bakerlab.org/index.php).

Although the method can be downloaded for local use, the equivalent CCMpred program [20] was used which provides a complete list of contacts, allowing some weaker inter-domain residue pairs to be considered. (General Public License v3 was downloaded from https://bitbucket.org/soedinglab/ccmpred).

#### Contact score

The accuracy of a predicted contact was evaluated as previously [18] using a ‘soft’ measure of contact based on either the separation of the $\alpha$-carbon atoms or the pseudo-centroids of the positions (as defined above), Both distances were inverted to give a score by a Gaussian function with an ideal separation of 10 Å for pairs of $\alpha$-carbon atoms or 6 Å for pseudo-centroids. (With an $\alpha$-carbon–centroid ‘bond’ of 2 Å, this is equivalent to the 10 Å $\alpha$-carbon separation). A spread factor (*c.f.* the standard deviation of the Normal distribution) of 5 Å was used for both types which means separations over 20 Å are effectively zero. Distances under the ideal separation were given a score of 1 (maximum) and those over 20 Å were ignored (score = 0).

The contact scores were typically plotted against the rank of the contact score but as this tends to be noisy, a cumulative score was plotted which is easier to assess visually. In a perfect selection, each pair will each score 1 so the closer this cumulative contact score approaches the line with unit slope (diagonal), then the better the result. This relationship was re-expressed as the gap between the score and the ideal line giving an error measure referred to below as the cumulative contact score error (CCSE), which should ideally remain zero over as many of the top-ranked contacts as possible.

### Investigation protocol

Each test protein was processed following the same protocol (or pipeline). A wider range of options and parameters were considered for those tested initially and if any appeared markedly detrimental they were not explored fully in later tests. For options that appeared to make little difference to the results, the simpler implementation of the method was adopted.

1. Domains were identified by a combination of manual and automatic methods [21].
2. The domain sequences were scanned against the sequences database, including Pfam, either separately or concatenated to gather between 5000–10,000 sequences for each domain (if possible).
3. The sequences from species that were found in all the alignments were extracted and classified as singleton or multiple entry.
4. The distance-based algorithm was applied to sequences belonging to a common species with tests being made varying the number of trial runs (10, 30, 50) and, to a lesser extent, the degree of noise added to the pairwise sequence distances (0.05, 0.1, 0.2).
5. The sequences for matched pairs of proteins were concatenated and the resulting joint alignment analysed for residue covariation.
6. The list of matched sequences was passed to the topology-based algorithm and the sequence matches recalculated. This was repeated varying the parameters as above with, in addition, variation in the number of outgroups (singletons) over 2, 4 and 6.
7. The results of both algorithms were analysed by the percentage of matched sequence identifiers and the fidelity of the predicted contacts, with the latter including a consensus over the runs with different parameter values. Varying degrees of re-ranking based on solvent exposure were investigated.

### Computer requirements

The two core algorithms differ in their execution times by around an order of magnitude. However, much of this difference is due to the inefficient execution of PHYLIP programs very frequently. If this component were recoded in a single program large savings would be made. The faster distance-based method is limited by the step of computing the distance of the current group of paralogs to the singleton outgroups. Limiting these to 50 (default) helps and while this number might be further reduced, time could be saved by precomputing all these distances (which are frequently reused). For these reasons, no algorithmic complexity estimates have been made for the algorithms.

In practical terms, the length of a calculation depends directly (in a linear manner) on the number of species and the number of random trials performed. Both of these aspects are completely independent of each other which means that all species and all random trials can be processed in parallel. Given, access to a compute-farm, the time for a run will be the time for one trial on one species. For the distance-based method, this currently takes on average one minute for a typical species with five paralogs in each of its three protein families.

## Results

Test data requires protein complexes of known structure, each component of which has sufficient sequences that can be aligned and equated with their partners across the protein families to generate a concatenated alignment. In bacterial systems, many such examples can be found and the alignments automatically generated and paired, for example by the GREMLIN method [16]. However, the automatic pairing across families retains an element of error which can be simply avoided by using domains in large proteins instead of subunits in a complex, which because of their covalent link through the protein chain, are known without doubt to coexist and interact in the same organism.

With an aim towards assessing the contribution of transitivity in the pairing process, proteins with at least three domains were selected with preference given to those that had compact, self-contained, structures including interactions between each pair of domains. For this the 3did domain database [22] (http://3did.irbbarcelona.org/) provides a valuable resource. A selection of proteins that met the structural requirements and also had sufficient sequences to generate an inter-domain correlation signal are listed in Table 1.

**Table 1:** Proteins used as test data

PDB	Protein	Length	Domain ends
		Used (full)	N–C (len)
1aoz	Ascorbate oxidase	434 (552)	9–126 (118)
			135–301 (167)
			378–526 (149)
1lci	Luciferase	404 (404)	24–186 (163)
			187–355 (164)
			359–435 ( 77)
1pkm	Pyruvate kinase	367 (390)	41–116 ( 76)
			117–388 (172)
			409–527 (119)
3ctz	Prolyl aminopeptidase	572 (617)	3–160 (158)
			161–319 (159)
			320–574 (255)
3vqt	Translation factor RF3	495 (495)	1–277 (250)
			278–389 (105)
			390–529 (140)
4rcn	Fatty-acid acyl-CoA carboxylase	401 (1972)	3–102 (100)
			103–345 (176)
			346–470 (125)

Against the protein name and PDB code, the length of the portion used is given with the length of the full chain in parentheses. The three domains used for each protein are specified by their residue numbers in the PDB entry and the length of the domain. (NB: because of missing segments in the PDB structure and omitted segments, the difference in the domain end-points does not necessarily equal the number of residues in the domain)

### 1aoz

Ascorbate oxidase, 1aoz, consists of three all-$\beta$ (Cu$^{++}$ binding) domains, roughly 150 residues in length, that mutually interact. Conveniently, there is a Pfam entry for each domain (PF07731, PF00934 and PF07732, in sequential order) but due to alignment profile shrinkage, each domain suffers some loss of sequence at their termini, however, the core domains and their interacting regions are unaffected.

**Fig. 2:**
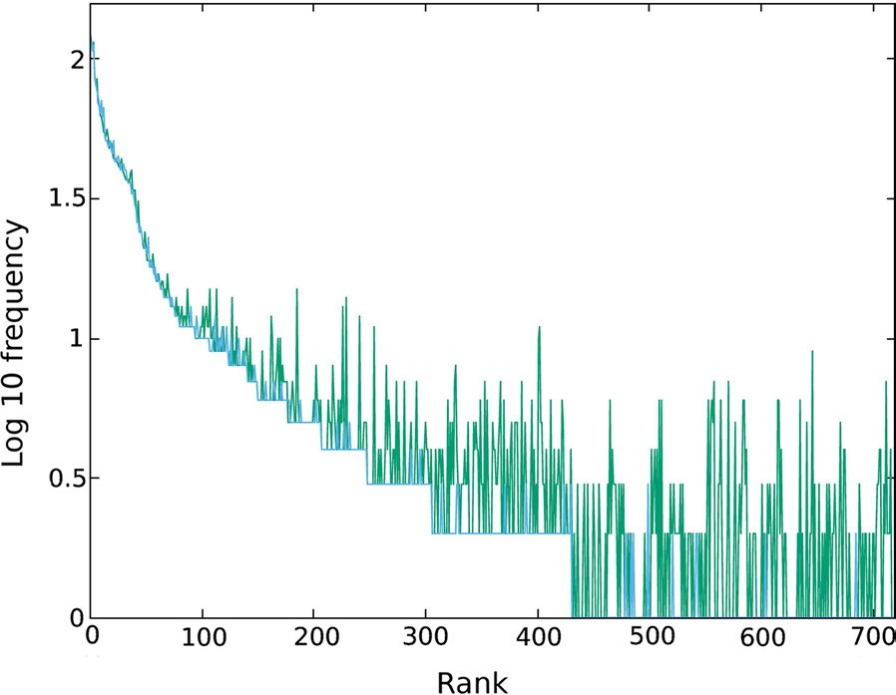
Sequence distribution per species. The number of sequences found for each of the 720 species is plotted (Y-axis as log$_{10}$) for each of the three domains that comprise the protein 1aoz (Ascorbate oxidase), ranked by the size of the smallest number of sequences in the three domains (X-axis)

Combining the Pfam families resulted in roughly 5000 sequences for each domain (Table 2). In which 720 species, were found in all three domain families and of these, 122 had a unique occurrence and were taken as the source of ‘outgroup’ species to combine with the subgroups of unassigned sequences. The distribution of the number of sequences for each species shows that about 85% have 10 or less sequences with the larger families rising to just over 100 members (Fig. 2).

#### Distance-based algorithm

Taking a cutoff of a maximum of 10 sequences per species and applying the tripartite matching algorithm to select a set of equal numbers of sequences for each species, reduced the total number of sequences for each domain to just under 2600, depending slightly on parameter choice. The two main parameters that were varied in the method were the number of trials and the degree of random perturbation applied to the edge-scores. These were evaluated by the percentage of correctly matched species names obtained over the three combinations of domains used in each test. However, this can only provide a rough absolute guide as close homologues may be selected which will still give a correct signal but will have different sequence identifiers. As such selections will be effectively random over a large number of species, the measure can, nevertheless, still be used for the relative comparison between runs. Based on this measure an average correspondence of 40% was obtained across all runs with little variation seen between parameter combinations. As the method appears quite insensitive to parameter variation, the randomisation factor was kept constant at 0.1 for all further runs while the number of trials was varied over the values 10, 30 and 50. The three values were retained to generate variation to allow a consensus selection of predicted contacts to be made.

**Fig. 3:**
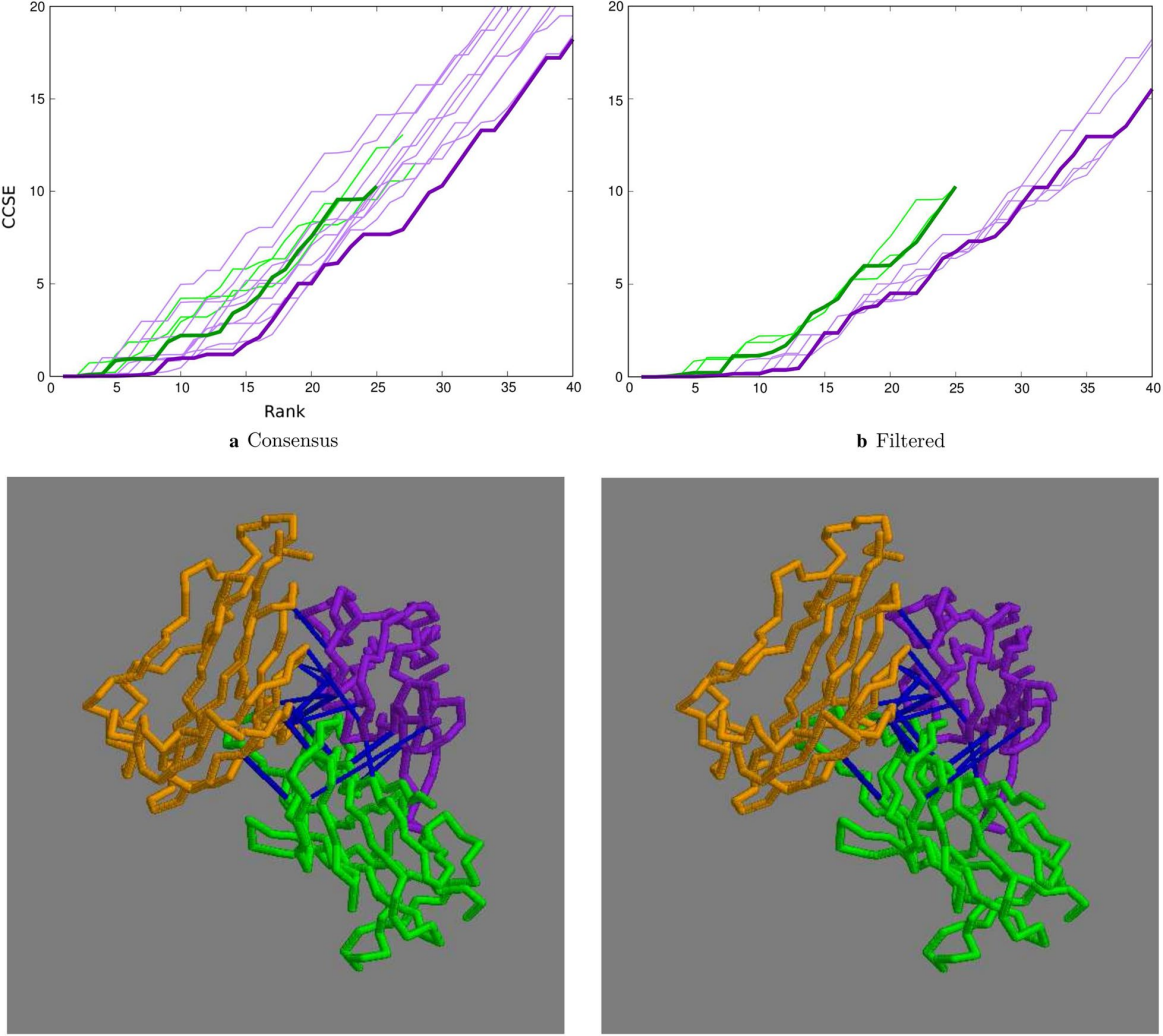
Top ranked contacts for 1aoz. **a** Raw data with the consensus plotted bold: green = distance-based, purple = topology-based. **b** Consensus plots filtered by residue exposure. Bold = 30% weight: green = distance-based, purple = topology-based. In both, the cumulative contact score error is plotted (Y-axis) against the rank of the pair (X-axis). The lower two panels constitute a stereo-pair of the structure with the domains coloured sequentially, purple, green, orange and the top predicted contacts linked by blue lines. The number of contacts to view was taken at the point where the purple line in (**b**) remained below a CCSE of 2

The concatenated domain alignments were analysed by the residue covariation method to generate predicted contacts and a visual assessment of the contact maps, as plotted by the GREMLIN server, revealed only two closely spaced inter-domain contacts, which were nonetheless correct. Examination of the extended list of contacts calculated by the similar CCMpred method indicated additional contacts, not all of which were correct. To estimate a possible lower cutoff to discriminate true from false contacts, the cumulated contact score error, or CCSE (see “Methods” section), was plotted against the rank of the pair. The CCSE for 10, 30 and 50 trials and their consensus remained low over the first five contacts, then rose gradually (Fig. 3a, thin green lines). Given that most residue pairs do not make any contact at all, this is clearly not a random selection.

To further reduce noise in the selection, only the residue pairs that were found in two or more datasets were plotted This reduced the error level over the top 15 contacts but gave little improvement in the top 5 (Fig. 3a, thick green line). There was little difference observed when requiring that each pair must be included in all three datasets. Adding the filter to reduce the weight of buried residue pairs resulted in a further improvement in selectivity with a 30% contribution having the greatest effect (Fig. 3b, green lines). The resulting selection of residue pairs would be sufficient to constrain the domains in a reasonably unique orientation but this will be evaluated more quantitatively elsewhere following the approach used previously for RNA [23].

**Fig. 4:**
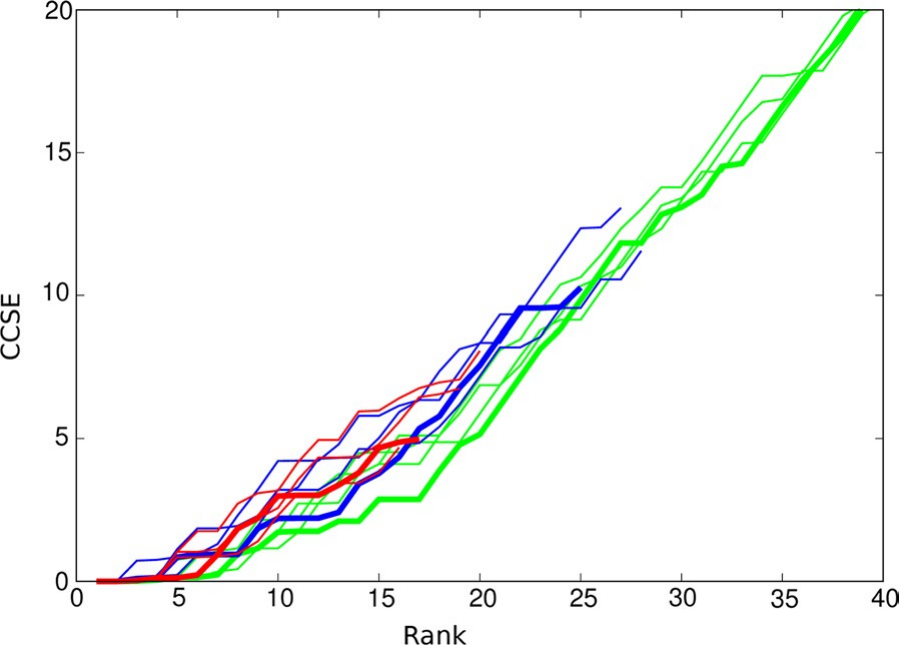
Top ranked contacts from the distance-based algorithm. The results of the distance-based algorithm with different maximum numbers of sequences per species are compared using the CCSE plot of the predicted contacts for a maximum of 5 (red), 10 (green) and 15 (blue) over three runs each with the consensus plotted with a thicker line. The corresponding sequence numbers were 1824, 2595 and 3015

With larger numbers of sequences per species, the chance of obtaining a correct pairing decreases but including more sequences should also help improve the correlation signal. This balance between sequence number and accuracy, which was set above at a limit of ten sequences per species, was reassessed using the CCSE plot. Limits of five and fifteen sequences per species were tested with the lower limit giving a 10% increase in accuracy and the larger limit a drop of 5%, both relative to the 40% accuracy obtained with the limit of 10. The corresponding sequence numbers were 1824, 2595 and 3015 and to see if this compensates for matching accuracy, the resulting cumulative contact score plot were compared (Fig. 4). These plots indicate that the ’default’ limit of ten species (green lines) gave the best selection of residue pairs and was retained as the default value throughout.

#### Topology-based algorithm

The phylogenetic tree (topology) based algorithm was tested as above but because of the greatly increasing computation time required for the permutation of the sequence order on the tree (see “Methods”), this approach was restricted to five unlabeled sequences embedded with up to six labeled sequences (the ‘outgroups’). The list of sequences produced by the distance-based method (which all have an equal number of sequences per species) was taken as input and the sequence pairings re-calculated. As above, the percentage of matching sequence identifiers was taken as a rough guide, followed by the comparison of the CCSE plots. In addition to the number of trials, the topology method has the further complication that the number of outgroups can be varied. As above, the results with 10, 30 and 50 trials were again evaluated but with each now in combination with 2, 4 and 6 outgroups.

**Fig. 5:**
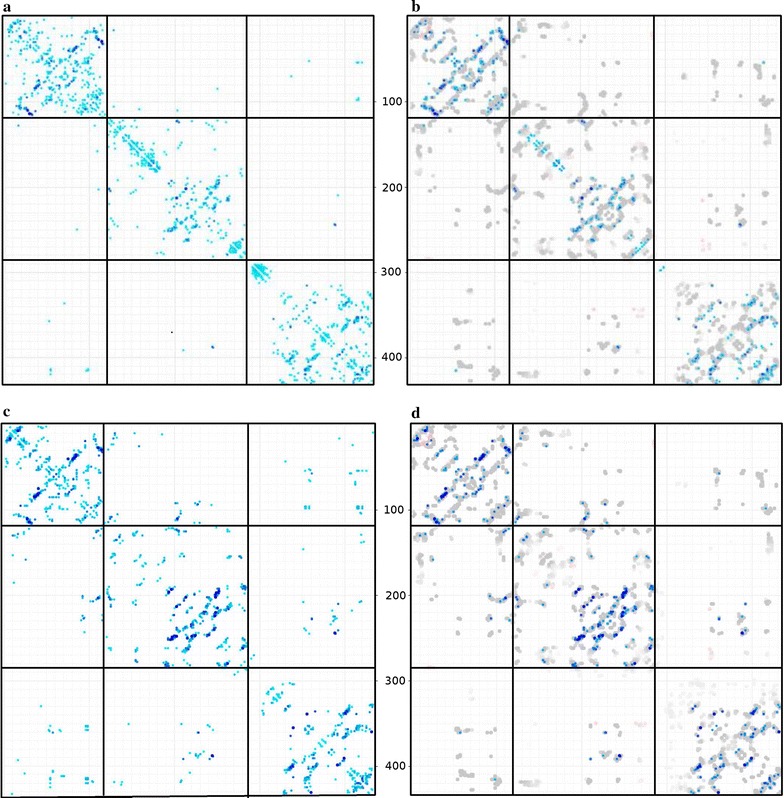
Contacts predicted by the GREMLIN method for 1aoz. The concatenated alignment generated by the topology-based method was submitted to the GREMLIN server with the results shown as a matrix of predicted contacts on a scale of light to dark blue (strongest). The origin is the top-left corner (**a**). In **b** the stronger contacts are overlayed on contacts from the closest matches found in known structures (grey dots), using an adaption of Taylor’s double-dynamic programming algorithm. For comparison, **c** and **d** show the contacts predicted by GREMLIN when given the full correctly paired concatenated alignment of domains. Bold lines correspond to domain boundaries and feint lines mark residues by 10 and 100s

The contacts predicted from the concatenated alignment have been visualised using the GREMLIN server which also uses the predicted contacts to find a structural match (Fig. 5). A clear correspondence in inter-domain contacts can be seen, even though some contacts lie towards the weaker end of the spectrum.

With a sequence identifier match of 58.6%, the topology algorithm was an improvement over the 52.6% obtained with the distance algorithm using the same upper limit of 5 sequences per species (averaged over all the runs). The nine CCSE plots for each parameter combination, along with their consensus plot show a corresponding depression (improvement) compared to the results from the distance algorithm (Fig. 3a, purple lines). With the addition of a small contribution from the SASA filter, up to 15 contacts remain relatively error free (Fig. 3b, purple lines, with the 30% contribution plotted as a thicker line). The contacts corresponding to this trace are shown in Fig. 3.

**Table 2:** Sequence data and pairing accuracy

PDB	Start	spec.	Number of seq.s			Percent code match		
			ones	dist.	topol.	dist.	topol.	no trans.
1aoz	8553	720	122	2588	982	39.7 (62.9)	58.6 (55.5)	54.8 (55.3)
	6356							
	7940							
1lci	5843	1579	565	3841	2045	63.8 (76.0)	75.7 (74.6)	60.8 (62.2)
	5830							
	5655							
1pkm	6243	1566	1026	3275	1989	72.3 (81.8)	80.0 (81.2)	79.9 (81.2)
	6499							
	6532							
3ctz	2095	634	487	1085	785	93.2 (94.7)	91.9 (91.5)	91.1 (90.8)
	2143							
	2137							
3vqt	4886	1132	633	2896	1858	95.8 (97.3)	91.3 (91.2)	91.6 (91.5)
	4886							
	4886							
4rcn	6018	705	120	2426	1151	50.2 (72.0)	53.1 (50.6)	53.1 (50.2)
	4212							
	5879							

For each protein (“PDB”), the number of sequences found for each domain in the initial databank search is tabulated under “start”, followed by the number of species common to all domains (“spec.”) and the number of species with a single sequence entry (“ones”). After processing by the distance-based algorithm the number of sequences common to all domains dropped (“dist.”) with a further drop on application of the more restrictive topology based algorithm (“topol.”). The rough measure of matching success, based on the identity of paired sequence codes is given for the two methods (“dist.” and “topol.”) as a percentage along with the success rate for the topology based method when the transitivity bias is omitted (“no trans.”). These values are averages over the three domain pairings but as these matches are not independent, the percentage over the first two domain pairs (1,2 and 2,3) are given in parentheses

#### Shuffled control test

As a control, the sequence pairings were shuffled. As there is no distinction between paralogs and orthologs at the sequence level, the shuffling was made at the final stage when the three domain alignments are concatenated. This was done by cyclicly permuting the sequence order in the second alignment by 1/3 and the third alignment by 2/3. This degrades the inter-domain contact signal to such an extent that, for example, almost all intra-domain contacts for the full 1aoz sequence (20,000) were ranked above the inter-domain contacts using the CCMpred program. As a stricter control, a similar shuffling was made just within each set of paralogs. This maintains the match of singletons and, on average, half of doubletons a third of triples, etc. As expected, these randomised results lie between the fully random results and the optimal results (see Additional file 1).

### 1lci

Luciferase, 1lci, has a core structure of three domains and a weakly interacting C-terminal domain which was removed. The remaining core domains, sequentially, two $\beta$/$\alpha$ domains followed by an all-$\beta$ domain, all interact closely and are sequentially separated.

**Fig. 6:**
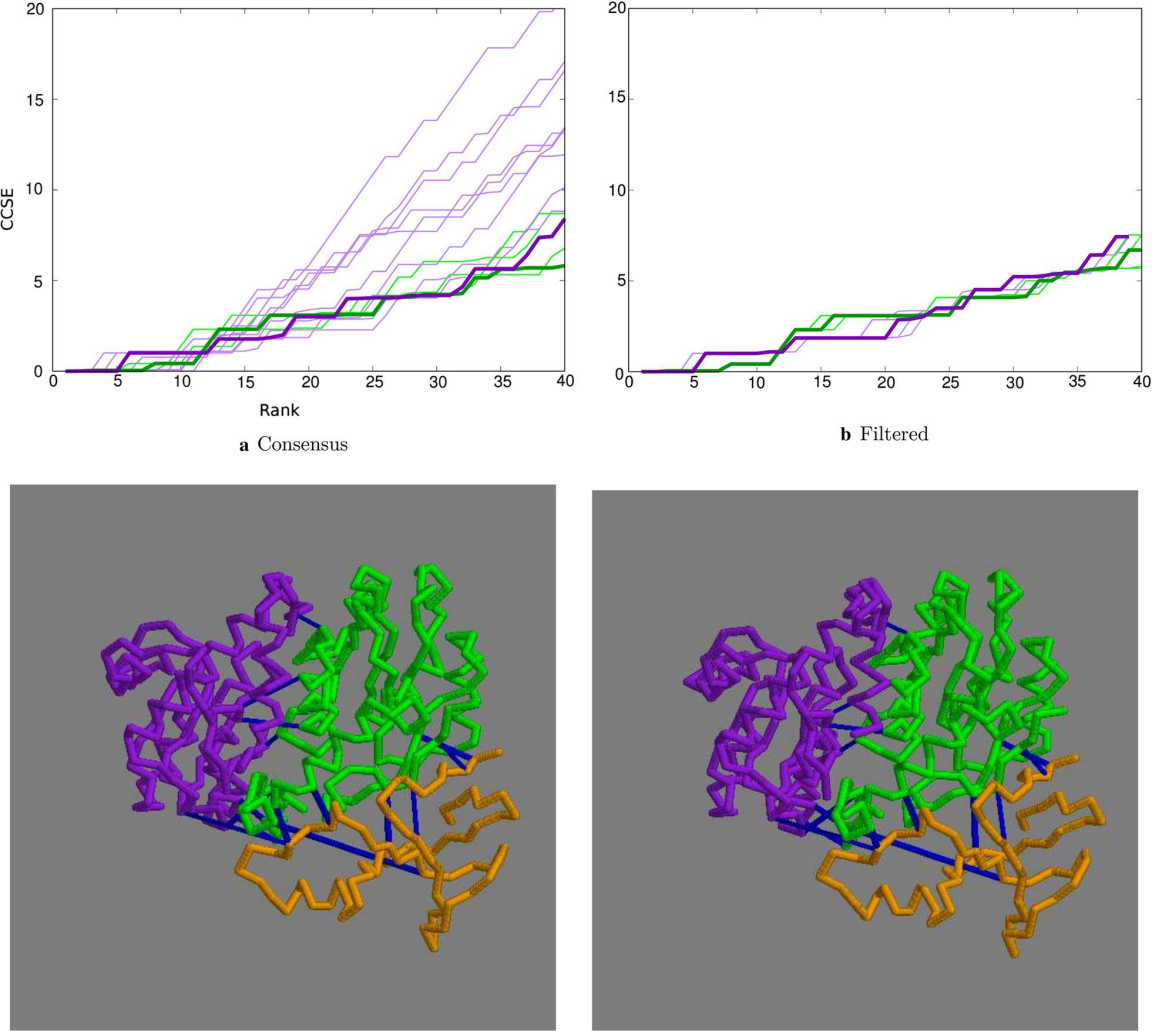
Top ranked contacts for 1lci. **a** Raw data with the consensus plotted bold: green = distance-based, purple = topology-based. **b** Consensus plots filtered by residue exposure. Bold = 30% weight: green = distance-based, purple = topology-based. The lower two panels constitute a stereo-pair of the structure with the domains coloured sequentially, purple, green, orange with the contacts in blue (selected as described in the legend to Fig. 3)

After reducing the initial databank sequences to those that had common species for each domain, 3841 remained, of which 565 were singletons. When matched with the distance-based algorithm, 64% correct sequence identifiers were matched (Table 2). In terms of contact prediction, this translated into a number of correct contacts (Fig. 6a, green lines). Taking the consensus, there were no errors in the top 6 and only one in the top 11 (Fig. 6a, thick green line).

Applying the topology-based algorithm to the set of 2045 sequences (reduced because of the lower upper limit of 5 sequences per species) resulted in a slight increase in the number of incorrect contacts but with the CCSE measure still remaining below 2 over the top 16 residue pairs (Fig. 6).

The SASA filter made little difference to either the distance-based or the topology-based results (Fig. 6b green and purple lines, respectively).

### 1pkm

Pyruvate kinase, 1pkm, has three distinct domains comprising a TIM barrel with a C-terminal $\beta$/$\alpha$ domain and an all-$\beta$ domain inserted roughly in the middle of the TIM barrel. As the inserted domain interacts less strongly than the other pair and the interruption in the TIM barrel makes the sequence manipulation more complex, this domain was removed and instead the two remain parts of the TIM barrel were treated as separate domains.

**Fig. 7:**
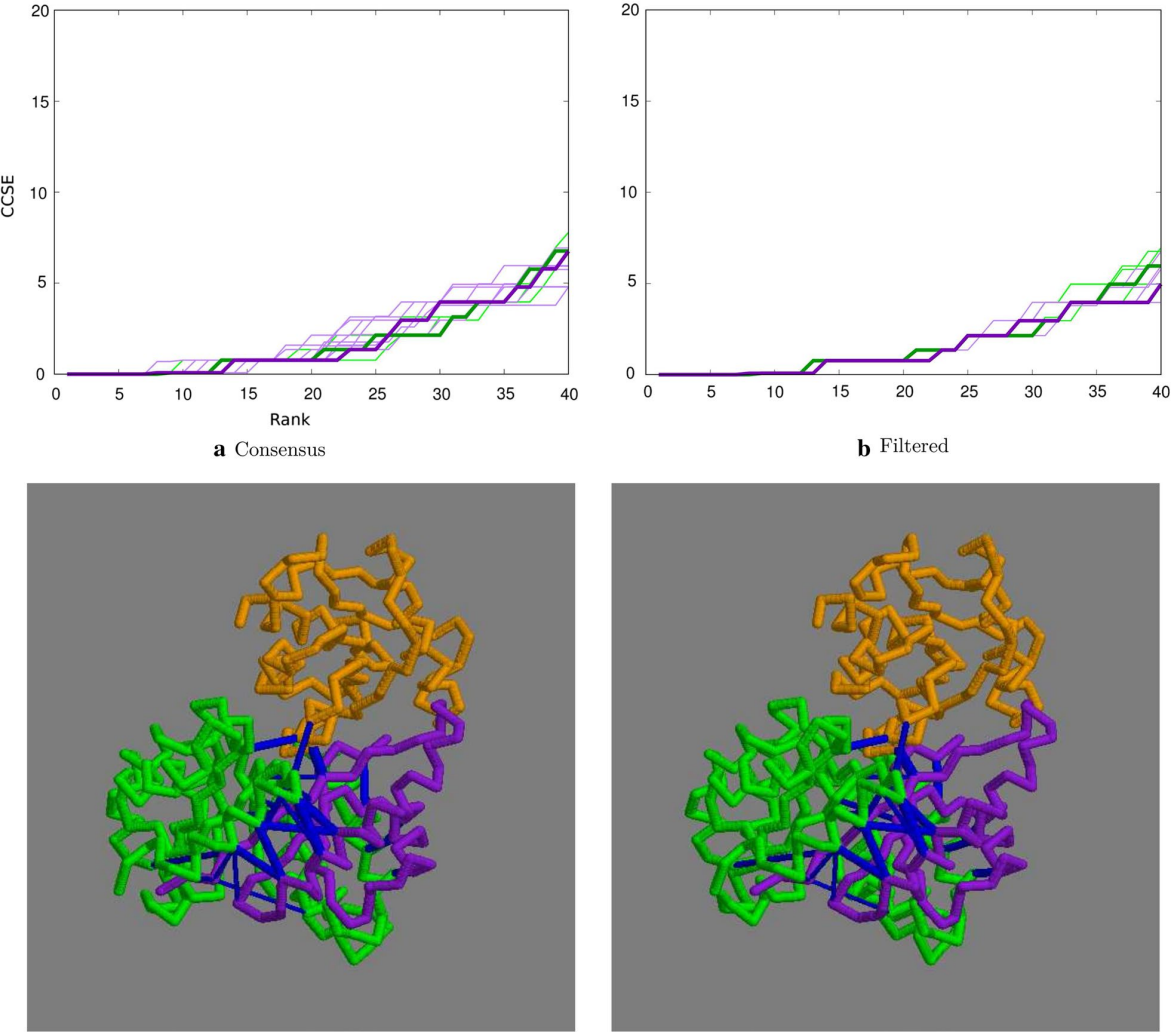
Top ranked contacts for 1pkm. **a** Raw data with the consensus plotted bold: green = distance-based, purple = topology-based. **b** Consensus plots filtered by residue exposure. Bold = 30% weight: green = distance-based, purple = topology-based. The lower two panels constitute a stereo-pair of the structure with the domains coloured sequentially, purple, green, orange and the contacts (blue) selected as previously

The initial 6500 odd sequences found for this protein dropped by half when filtered for common species and of the remaining 3275, 1026 were singletons. Boosted by this large number of correctly matched singletons, the overall percentage of matched sequence identifiers was 72% (Table 2). This translated into well predicted residue pairs with errors only starting to accumulate after the top 20–30 contacts (Fig. 7a). The SASA filter gave little improvement with either the distance- or topology-based methods (Fig. 7b).

The majority of the top predicted contacts were between the first two domains which were the parts of the TIM barrel. However, within the top 20 contacts, three good links were seen between the third domain and the previous two (Fig. 7).

### 3ctz

Cytosolic X-prolyl aminopeptidase, 3ctz, has three interacting $\beta$/$\alpha$ domains, with the largest C-terminal domain terminating in two $\alpha$-helices that do not contribute to the domain interfaces which were removed.

Despite the small number of just 1085 sequences after filtering and 487 singletons, the percentage of matched sequence codes was remarkably high at 93%. This appeared to be due to the lower number of sequences reducing the number of sequence per species to more managable groups with almost all falling under five.

**Fig. 8:**
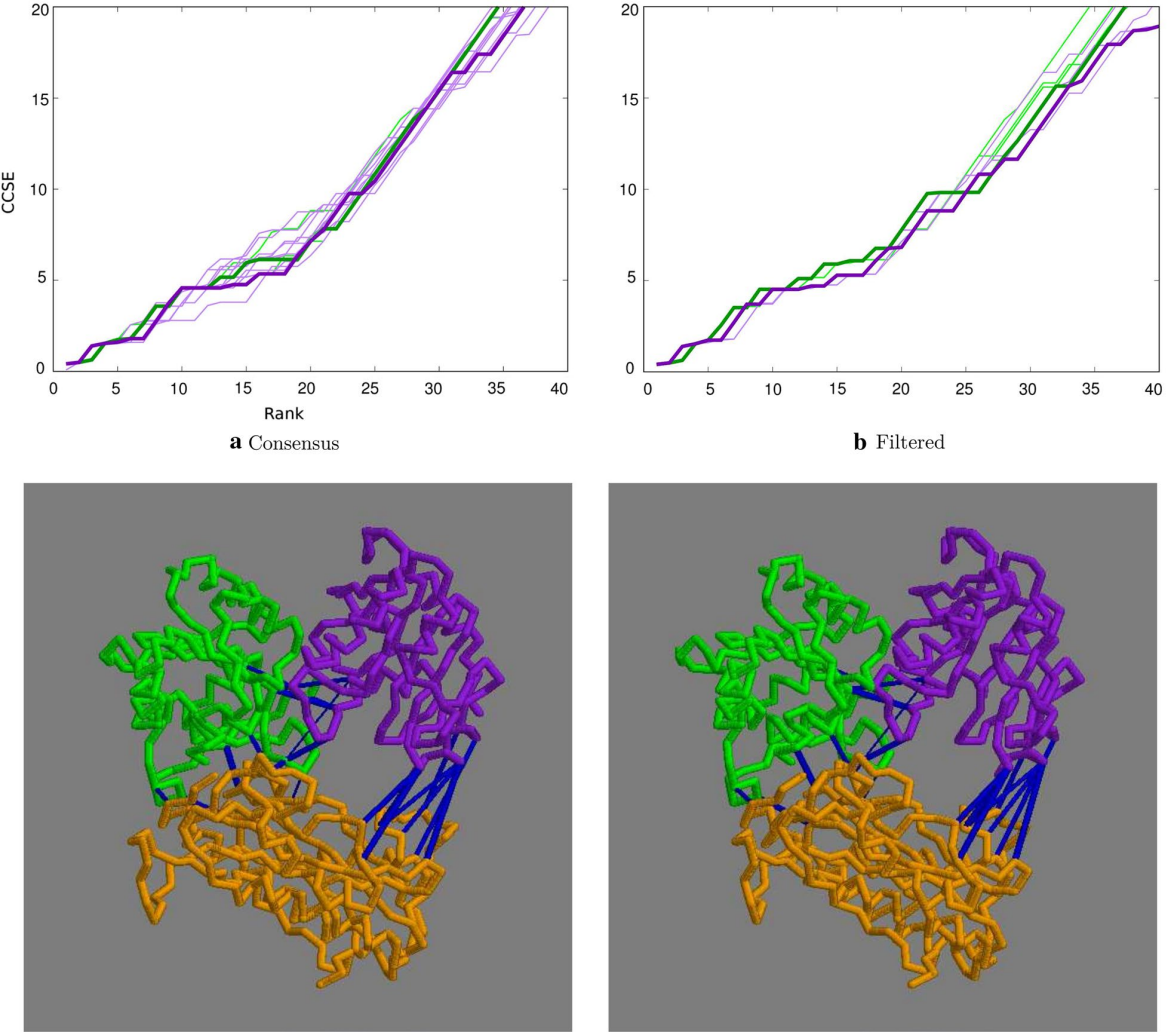
Top ranked contacts for 3ctz. **a** Raw data with the consensus plotted bold: green = distance-based, purple = topology-based. **b** Consensus plots filtered by residue exposure. Bold = 30% weight: green = distance-based, purple = topology-based. The structure is a stereo pair with domains coloured sequentially, purple, green, orange and contacts in blue (selected as described above)

The resulting predicted contacts, which were similar for both methods, gave an unexpectedly high CCSE (Fig. 8a) which was not helped by any degree of exposure filtering (Fig. 8b). Examination of the contacts on the structure (Fig. 8) clearly showed that the top 15 contacts were effectively correct but those between the first and last domains were long, up to 20 Å. It seems likely that these constitute a meaningful covariation signal, either originating through interaction with a common moiety, or more likely, come into contact through domain movement in the the native protein.

### 3vqt

The translation factor RF3, 3vqt has three distinct, closely interacting, domains that were adopted without editing.

**Fig. 9:**
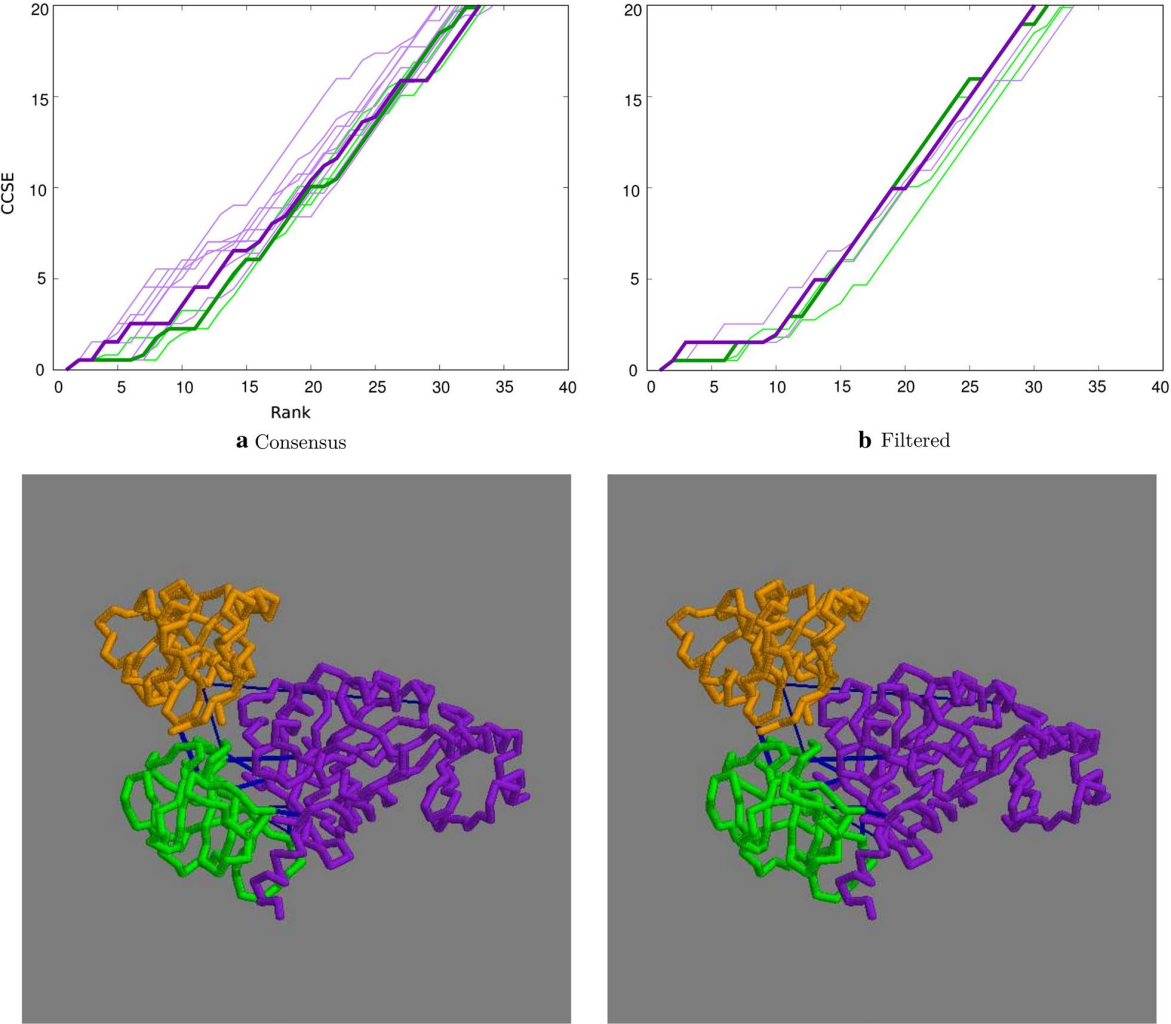
Top ranked contacts for 3vqt. **a** Raw data with the consensus plotted bold: green = distance-based, purple = topology-based. **b** Consensus plots filtered by residue exposure. Bold = 30% weight: green = distance-based, purple = topology-based. The structure has domains coloured sequentially, purple, green, orange with contacts in blue (selected as described above). Unlike the previous examples, the top contacts predicted by the distance-based algorithm are shown

The initial run of the distance-based algorithm gave a remarkably accurate pairing of sequence identifiers of 96% over almost 2900 sequences (of which 633 were singletons). Despite this accuracy over a good number of sequences, only the top 7 contacts were correctly predicted by the distance-based method and even less with the topology-based method, before errors quickly accumulated (Fig. 9a). A situation that was not helped by any degree of SASA filtering (Fig. 9b).

### 4rcn

The long-chain fatty-acid acyl-CoA carboxylase, 4rcn, is a large multi-domain protein but has an amino terminal group of three mutually interacting domains.

**Fig. 10:**
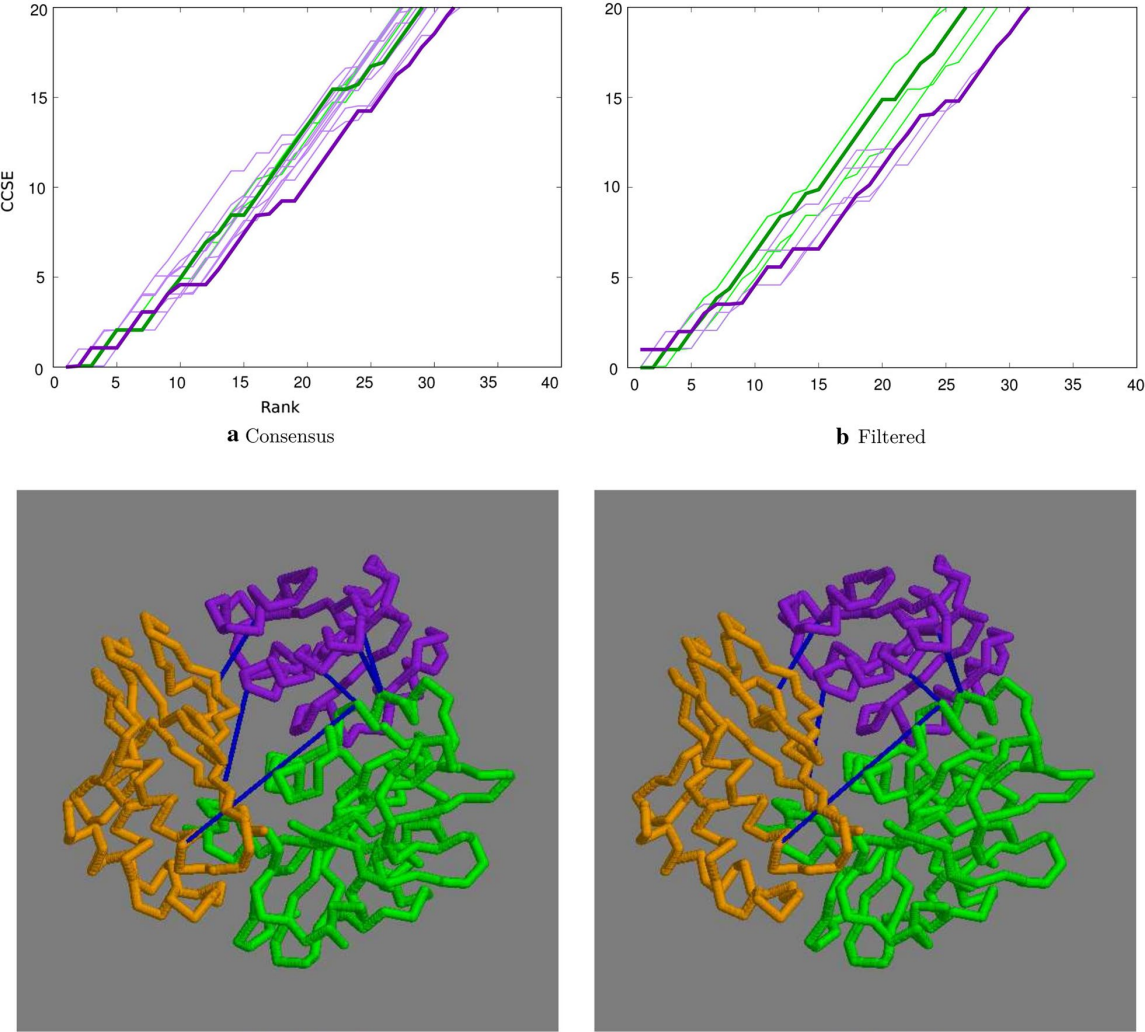
Top ranked contacts for 4rcn. **a** Raw data with the consensus plotted bold: green = distance-based, purple = topology-based. **b** Consensus plots filtered by residue exposure. Bold = 30% weight: green = distance-based, purple = topology-based. The structure has domains coloured sequentially, purple, green, orange with contacts in blue (as above)

Despite having a typical distribution of sequences and sequence codes matched to a comparable degree as 1aoz, the predicted contacts for this molecule were the poorest yet observed. With no apparent reason for this in the distribution of data, it may be the result of errors in the data such as misaligned sequences (as the full sequence is very long), or simply due to natural variation in the degree to which the domains interact (Fig. 10).

### Transitivity contribution

The contribution of consistent pair selection (transitivity) was assessed by removing the bonus score given to transitive relationships. It can be seen from the percentage of matched sequence codes that this can make a marked contribution to those that have a lower percentage but when the quality of match is high, the contribution is not noticeable (Table 2, rightmost column).

Although the boost to select transitivity takes no additional computer time, it does reduce the number of sequences by requiring that there is sequence member present in each of the three proteins. To test this effect, a sample of the proteins considered above were processed as domain pairs and it was found that the numbers dropped only by between 10 and 100. These were run as pairs and to maintain comparability this test used the same code but with two of the three domains being identical. This ‘short-circuits’ the transitivity test causing it to default to a test for reflexive relationships that were still given the same bonus when they were found. On a limited number of trials, the results were not significantly different from the triple sets within the ‘noise’ level of repeated runs (data not shown).

It should be noted that the domain derived examples considered above have well balanced sequence sets since the three domains are derived from the same chain. If the sequences had been collected independently, a larger difference would be expected. In this situation, running both as domain pairs (as in some additional examples below) and as triples (to gain any transitivity contribution) would be recommended. The results from these differing runs can, of course, still be pooled into a consensus as described above.

### Cutoff selection

All the methods and filters tested above give no indication of the extent to which predicted contacts can be trusted. The degree of error that can be tolerated will, of course, depend to some extent on how the information is used but assuming that some form of docking method will be employed to satisfy the predicted contacts as restraints, then it is likely that more than 30% error would make it difficult to find a unique solution.

Three approaches to this problem are explored below using internal consistency tests based on aspects of the known structures of the component domains, including the situation where the interaction of one pair of domains is known.

#### Exposure based cutoff

The principle that buried contacts should be less likely to contribute to an interface was tested above as a filter on the selection of residue pairs. Although the benefit of this filter was marginal, it was re-tested as a criterion on which to limit the number of contacts that should be considered. A measure of burial error was taken as $1-w$, where *w* is the Gaussian transform of the product of the SASA for a pair of residues (see “Solvent exposure filter” section). This score is 1 for a fully exposed pair and 0 for a completely buried pair. The score was accumulated over the ranked list of residue pairs in the same way as the CCSE.

**Fig. 11:**
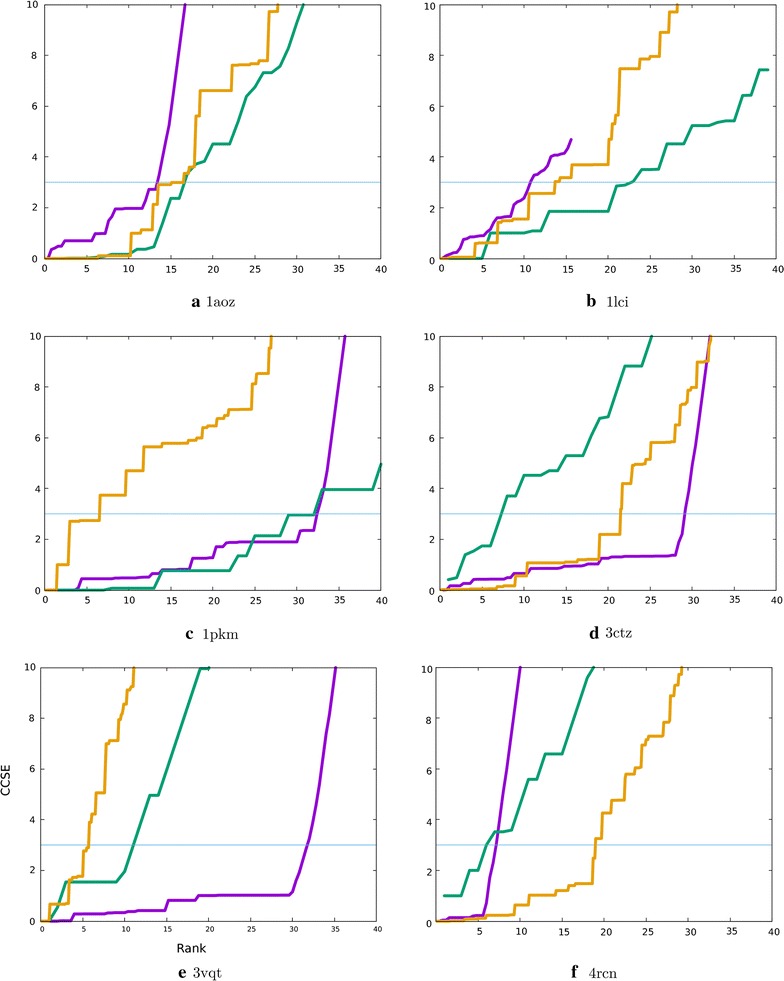
Internal controls on pair numbers. Two internal quality control measures were evaluated to assess pair selection based on the known domain structures. The CCSE score for inter-domain residue pairs (green) is plotted along with the CCSE score for intra-domain pairs (orange) but with the rank of the latter (X-axis) scaled by a factor of 1/10 to make the plots commensurate. The accumulated burial score for inter-domain residue pairs is also plotted with a smaller reduction by 1/2 (purple)

The plot of the accumulated burial score was generally low for the top ranked residue pairs (in agreement with their expected surface location) and rose at a rate of roughly half that observed for the CCSE. Applying a scale factor of 0.5 to the rank, allowed these two measure to be compared more easily (Fig. 11, green line = CCSE, purple = burial error). For four of the proteins (1aoz, 1lci, 1pkm, 4rcn), the curves run reasonably close, by contrast, a substantial shift to the right (higher rank) was seen for 3ctz and 3vqt. If taken as a cutoff, this would lead to the inclusion of too many contacts. However, this would be a reasonable outcome for 3ctz where the long contacts between domains 1 and 3 can be considered correct but for 3vqt false contacts would be included.

#### Inter/intra contact balance

In the test examples used above, only the contacts between domains were considered, however, the covariation analysis gives predictions for all pairs of residues and as the structures of the component domains are assumed to be known, then the increase in internal contact errors can be used to identify a cutoff limit. Because of their weaker degree of interaction and the errors in sequence matching, the inter-domain contacts will be predicted with a much lower score than their internal counterparts. Thus a relative comparison is needed.

The error in the intra-domain contacts accumulated much more slowly than the inter-domain contacts (for the reasons mentioned above) and applying a factor of 1/10 to the rank scale brought the majority of the curves closer to the inter-domain CCSE curve (Fig. 11, orange lines). An exception was 1pkm, which is dominated by the contacts between the two halves of the TIM barrel which are effectively internal contacts which suffer little loss of fidelity because of the accurate matching of sequence pairs (Fig. 11c). For 3ctz the intra-domain contact error plot, like the burial error plot, also stayed low over more of the ranked list of residue pairs indicating, correctly, that more contacts should be included than would be suggested by the CCSE plot (Fig. 11d). Unlike the burial measure, for 3vqt the internal contacts indicated that a stricter cutoff should (correctly) be applied (Fig. 11e) but for 4rcn the situation was reversed with the internal contacts (wrongly) admitting more residue pairs than the burial measure.

#### Consensus cutoff

Combining the two measures evaluated above may lead to a more robust cutoff and simply taking the point where the average of the two curves equals 3 (feint blue line on the plots in Fig. 11) would give a good estimate of the number of correct contacts. However, such is the variation between individual proteins that more examples will need to be tested.

### Known pair interaction

The test examples used above were chosen to have three interacting domains not only to test the benefit of finding transitive relationships but also so that the interaction of a pair of domains can be assumed to be known and used as a way to set a limit on the number of predicted contacts to be considered.

This was evaluated by finding the limit in the ranked residue pairs within which all predicted contacts between domains 1 and 2 were correct. The same number were then evaluated between domains 1 and 3 and between domains 2 and 3 using the criteria that the resiue pair must be within the 20 Å cutoff, with a clear ’view’ of each other. (This includes the widely separated residue pairs between domains 1 and 3 in 3ctz.) A simple table of true and false tally of contacts was then compiled (Table 3).

**Table 3:** Predicted contact numbers assuming one known pair

PDB	Domains 1,2	Domains 1,3		Domains 2,3		Score
	(Known)	T	F	T	F	
1aoz	3	3	0	3	0	6/6
1lci	10	0	2	8	1	8/11
1pkm	11	3	7	4	6	7/20
3ctz	7	7	0	6	1	13/14
3vqt	6	1	5	2	4	3/12
4rcn	3	1	2	2	1	3/6

For each protein (“PDB”) the maximum number of correct contacts between domains 1 and 2 (“known”) was used to limit the residue pairs considered between the other domain pairs. In these interactions, the number of true (“T”) and false (“F”) contacts is tabulated and scored by the fraction correct (“score”)

Although this is only a limited and superficial evaluation, it is sufficient to high-light pit-falls. Most obviously, from the example of 1pkm where it is clearly not useful to use the contacts in an artificially split single domain (the TIM barrel) to set a limit on less tightly interacting domains—even if both have had their sequences paired in the same way. However, with the exception of 3vqt, a reasonable selection was made for the other proteins with most scoring 50% and over.

### Comparison to other methods

The two methods, mentioned in the Introduction [4, 5], that directly maximise the strength of the predicted contacts are evaluated below. Given limitations on computer time, this comparison is not an exhaustive benchmark but is sufficient to give a clear indication of the relative performance of the methods.

#### Bitbol et al. method

Bitbol et al. tested their method on the bacterial histidine-kinase/response-regulator system. The kinase is a large protein and the regulator is a small protein of which che-Y is a typical family member. There is no shortage of sequences in both families, which in the current Pfam databank, have 85,578 and 176,760 members in the “full” entry or around six times as many in the “Uniprot” and “NCBI” entries in the database. Not unsurprisingly, the authors did not deal with these numbers but used around 5000 sequences from each family and a fragment of the kinase comprising the alpha-helical hairpin that acts as a ’docking-platform’ for the response-regulator. Running on a single laptop, it was practical to run this system with the parameter $N_{increment}$ set to 25 which gives around 80% accuracy in pair assignment. Such a run takes several hours.

The same dataset when processed using the current distance-based method gave 70.8% accuracy over 654 sequence pairs with the family size limited to 5 paralogs or 50.6% over 1900 sequence pairs with a limit of 10 per family. As it was suspected that these results were hampered by the small size of the kinase fragment, the full topology-based method was not run and the proteins were rerun instead using the full protocol outlined above starting with the smaller Pfam entries from the reference proteome datasets (rf15) comprising 15,502 and 31,596 sequences extracted from the families PF00512 and PF00072, respectively. With these, still large datasets, the current method was only run using the distance-based methods which is sufficient to provide a lower-bound on performance. This gave a percentage accuracy of pair assignment of 72.2%, but only over 306 sequence pairs. As shown above, following this with the topology-based method is likely to add up to 10%. Despite the large number of starting sequences, the low number remaining most likely is because the rf15 datasets have been independently reduced for each protein without the requirement to preserve matching species.

These limited results provide an indication that, although the current method falls behind on the Bitbol et al. test dataset, it retains the capacity to be applied to larger systems with equal effect, but further testing should be better carried out using protein families of a more managable size.

#### Gueudré et al. method

Gueudré et al. also tested their method on the histidine-kinase signaling system as well as proteins from the bacterial tryptophan synthesis operon, comprising the genes trpA..G, of which trpA/B and trpE/G correspond to interacting protein pairs. Given the similarity of the two methods, the trp-synthase A/B pair was selected for comparison, which is also conveniently provided as the test example in their download (from https://github.com/Mirmu/ParalogMatching.jl). As with the previous method, these sequence data are fragments (under 50 residues) and would not be suitable test data for the current method. As above, the Pfam families for trpA (PF00290) and trpB (PF00291) were considered, but having 4195 and 32,960 sequences, respectively, the more balanced selection of 4139 and 6968 (after removal of close homologues) returned by the GREMLIN server was preferred. These alignments were generated using the sequences from the complex of known structure (PDB: 1k7f) as a probe, allowing the inter-chain results to be directly evaluated over this structure.

Having now lost the reference pairings from the original test-set, a simple check was made on pairing accuracy by counting only sequence pairs from species in which the proteins were labeled “TRPA” and “TRPB”. This gives a random sample of around 50 sequence pairs which is sufficient for a rough comparison. By this measure the current method made 49 pairings of which 75.5% were correct (run with max.10 parlogs) while the Gueudré et al. method found 52 with 73.1% correct. In addition, contacts from the concatenated alignments were predicted and visualised by GREMLIN but both methods found almost no inter-protein contacts despite their reasonable pairing accuracy. In the top 2000 predicted contacts, the current method had one (wrong) inter-protein contact prediction while for the Gueudré et al. method, one predicted contact in the top 5 was correct (with 3 in the top 10). Although the current method may do slightly better if using the topology-based approach, both methods fall far behind what can be obtained using gene co-location (see: http://gremlin.bakerlab.org/cplx.php?uni_a=P0A877&uni_b=P0A879).

**Fig. 12:**
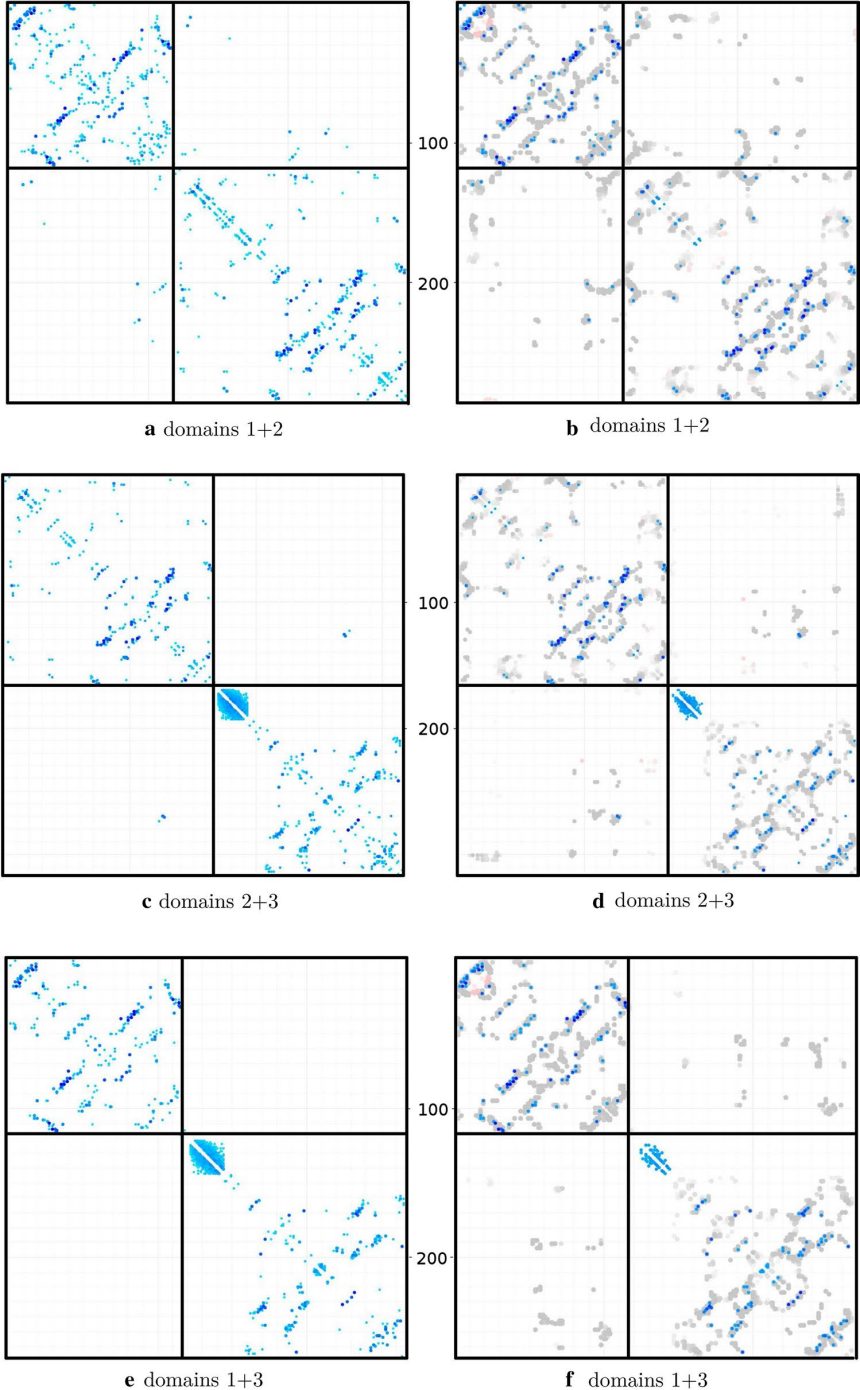
Contacts predicted by the GREMLIN method for 1aoz. The concatenated alignment generated by the method of Gueudré et al. was submitted to the GREMLIN server with the results shown as a matrix of predicted contacts as in Fig. 5. Three pairs of plots are shown for the three domain combinations. Bold lines correspond to domain boundaries and feint lines mark residues by 10 and 100s

The Gueudré et al. method was also tested on the three domain pairings from 1aoz using the alignments generated above. As the domain sequences each retain the original code of the protein, their pairing accuracy can be measured unambiguously over all sequence pairs. This gave percentages of 37.7, 38.8 and 43.9 over domain pairs 1,2 and 2,3 and 1,3 with 6246, 6269 and 7643 sequences respectively, which can be compared to the 58.6% obtained with the current method but over a smaller number of sequence pairs. Again, the concatenated alignments were used to predict contacts in GREMLIN where some inter-domain contacts are predicted between adjacent domains by the Gueudré et al. method but none between domains 1 and 3 (Fig. 12). This can be compared to those found by the current method where contacts are predicted between all domain pairs (Fig. 5).

## Discussion

### Summary of the results

The methods were tested on a set of proteins that covered a wide range of situations. Despite all having a sufficiently large initial collection of sequences, the initial matching of sequence pairs ranged from 40% of correctly paired identifiers to over 90%. When translated into predicted contacts, the order of success was not preserved, probably due to the relatively arbitrary choice among close homologues causing a loss of matches when only sequence code names were compared. Indeed the worst protein (1aoz) and the best protein (3vqt) as measured by code matching had almost swapped positions when assessed by the quality of their contact predictions. There appeared to be no correlation in the quality of the final prediction with either the number of sequences (as long as there were sufficient to produce a signal), the number of species or the number of singletons (one sequence per species). Adding a bonus for transitivity had some benefit for those with a lower percentage of matched codes but, obviously, did little to improve pairings that are already almost perfect.

The use of solvent accessible surface area to filter the selected pairings also had some benefit but this was less than might have been expected. This may have been because direct contact is not an essential requirement to generate a correlation signal between a pair of residues but more probably, is simply a consequence that in small domains, there is a large number of exposed residues relative to those that are completely buried, so giving less selection power.

The application of residue burial to the difficult problem of setting a cutoff to the number of predicted contacts to accept as true was, however, more successful and when a cumulative error based on the inclusion of buried residue pairs was monitored, in most cases, it provided a good guide to the point at which errors rapidly accumulate. The addition of the CCSE score based on predicted internal domain contacts also had useful information and between them the two scores might form the basis of a cutoff measure.

### Algorithmic evaluation

The two algorithms developed in this work, distance-based and phylogenetic-tree or topology-based, were originally viewed as a main method (topology) fed by a pre-filter based on distance. However, the good performance of the distance-based measure itself cast this relationship into doubt. Although the tree-based measure remained superior, in its current implementation it is limited by the burden of comparing many trees generated by combinatorial enumeration. Much of this burden could be eliminated by avoiding the use of repeated calls to external programs from the PHYLIP package but, in principle, the combinatorial ‘explosion’ for larger numbers of sequences-per-species will remain a barrier even if shifted slightly to allow greater numbers to be considered. It is possible that improvements to the distance-based algorithm may provide the more hopeful path for development, such as introducing a partial tree structure in the form of a minimal-spanning-tree (MST), however, there are currently no suitable MST comparison algorithms for trees with partially labelled nodes.

Although the method was only tested with double and triple domain/subunit proteins, this is not a fundamental limitation. Extending to larger numbers of domains (and/or subunits) could be implemented by extending the test for transitive relationships to a test for cyclic braids. While this may be implemented in the future, as suggested in the introduction, a simpler extension could be made with the current code by running sets of overlapping triples.

### Conclusions

The methods developed here have been designed to solve the problem of matching paralogs from a pair of proteins within a single species without using any known information about those proteins except their amino acid sequence. The method is aimed at eukaryotic proteins where the number of sequences remains low compared to bacterial proteins and the numbers of sequences used in this work were kept low to reflect that limitation.

Both derived information in the form of predicted contacts and external information, such as gene location, can be used in addition to find solutions to the sequence matching problem. The power of maximising predicted contacts was evaluated above and found to be comparable to the current approach but as both methods use unrelated algorithms it might be hoped that a joint analysis would lead to additional improvements. Being able to work on relatively long sequences also would allow the current method to pre-process the data to focus the more time-consuming distance calculations towards a probable interaction zone.

External information can be found in the form of functional annotation and protein-protein interaction networks as well as gene location in the genome, which is used to good effect in the simpler bacterial genomes. As gene co-location in eukaryotes seems to be limited to occasional tandem duplications [24], methods such as the SNAP algorithm [25], might be used to extract less obvious gene location relationships. Development of the current method will consider these sources.

## Additional file

**Additional file 1: Figure S1.** CCSE plots of randomised controls. The results of the topology (tree) based method shown in purple as in the main text (eg: Fig. 3a). Fully shuffled matches are plotted in blue and matches shuffled only within a family are plotted in green. The latter preserve the identity of singleton families and doubletons with 50% chance, etc. Note that 1pkm, which included a split domain and has many singletons still scores well and that for 3ctz, the purple lines run higher than expected because of the longer predicted links between domains 1 and 3 (see Fig. 8).
