# Supplementary material for: Algorithms for matching partially labelled sequence graphs
Source: Algorithms Mol Biol. 2017 Sep 25;12:24. doi: 10.1186/s13015-017-0115-y (PMC5613400; doi:10.1186/s13015-017-0115-y)
Supplement: Supplementary file 1 — Additional file 1: Figure S1. CCSE plots of randomised controls. The results of the topology (tree) based method shown in purple as in the main text (eg: Fig. 3a). Fully shuffled matches are plotted in blue and matches shuffled only within a family are plotted in green. The latter preserve the identity of singleton families and doubletons with 50% chance, etc. Note that 1pkm, which included a split domain and has many singletons still scores well and that for 3ctz, the purple lines run higher than expected because of the longer predicted links between domains 1 and 3 (see Fig. 8). [file 13015_2017_115_MOESM1_ESM.pdf]

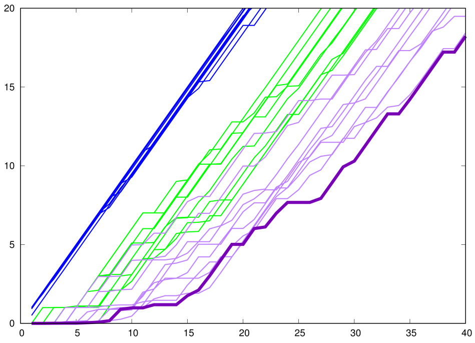

(a) 1aoz

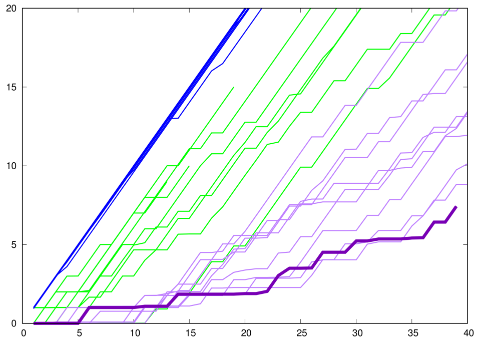

(b) 1lci

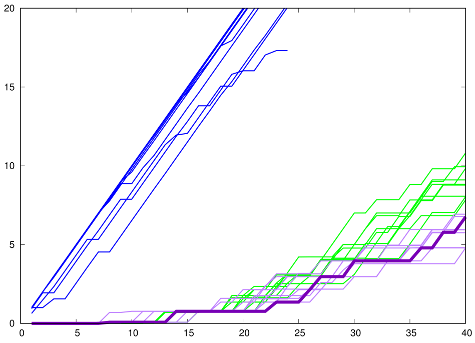

(c) 1pkm

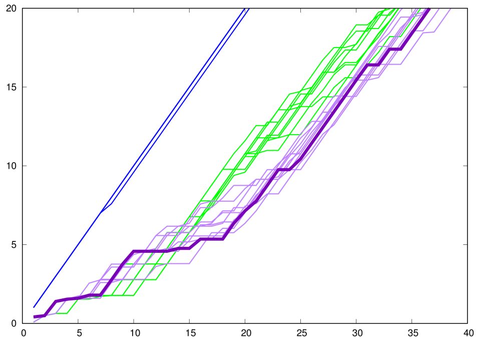

(d) 3ctz

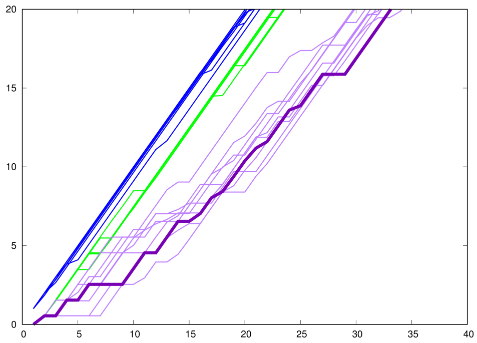

(e) 3vqt

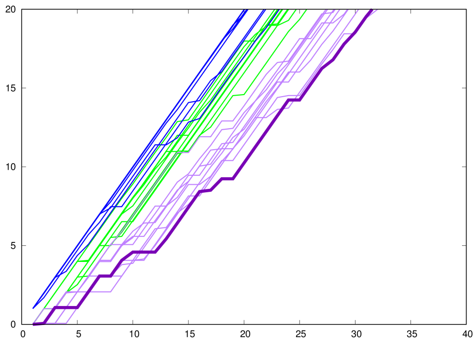

(f) 4rcn

Figure 1s: **CCSE plots of randomised controls**. The results of the topology (tree) based method are shown in purple as in the main text. (eg: Figure 3a). Fully shuffled matches are plotted in blue and matches shuffled only within a family are plotted in green. The latter preserve the identity of singleton families and doubletons with 50% chance, etc. Note that 1pkm, which included a split domain and has many singletons still scores well and that for 3ctz, the purple lines run higher than expected because of the longer predicted links between domains 1 and 3. (See Figure 8).
